# Ovarian cyst elevation using a metreurynter for laparoscopic cystectomy of a benign ovarian cyst during pregnancy

**DOI:** 10.1186/s12884-021-03774-w

**Published:** 2021-04-23

**Authors:** Yasushi Kotani, Kosuke Murakami, Kiko Yamamoto, Risa Fujishima, Tamaki Yahata, Yoshie Yo, Masao Shimaoka, Noriomi Matsumura

**Affiliations:** Department of Obstetrics and Gynecology, Kindai University Faculty of Medicine, 377-2 Ohno-higashi, Osaka-sayama, Osaka 589-8511 Japan

**Keywords:** Laparoscopic surgery, Metreurynter, Pregnancy, Ovarian cyst elevation, Ovarian tumor

## Abstract

**Background:**

A uterine manipulator cannot be used to elevate the ovary in benign ovarian surgery during pregnancy. This report describes our method of elevation of the ovary using a metreurynter with the success rate of the procedure and a comparison of surgical results and pregnancy outcomes between the successful and unsuccessful cases.

**Methods:**

Between August 2003 and February 2020, 11 pregnant patients with a tumor found sunk in the Cul-de-sac underwent laparoscopic cystectomy for a benign ovarian cyst with a metreurynter. The surgical results, success and failure of the elevation by a metreurynter, pregnancy outcomes, and fetal status at delivery were evaluated.

**Results:**

Elevation of ovarian tumors with a metreurynter was successful in nine cases. However, it was unsuccessful in the remaining two cases wherein the ovary was lifted with forceps while the uterus was in a compressed state. The operative time was also longer in these cases. The pregnancy prognosis, however, was good for both, successful and unsuccessful cases.

**Conclusions:**

The metreurynter is an inexpensive and practical obstetric device, and its optimal use allows the performance of a procedure with minimal burden on a pregnant uterus. Therefore, we recommend the appropriate use of this method to enable effective laparoscopic cystectomy of ovarian tumors during pregnancy.

## Background

Ovarian tumor is one of the most common gynecological tumors. Most ovarian tumors are asymptomatic and detected for the first time on ultrasonography. Benign ovarian tumors reportedly complicate 5–6% of all pregnancies [1]. Because it is less invasive than open surgery, laparoscopic surgery is currently the gold standard for the treatment of benign ovarian tumors in non-pregnant women [2]. However, the effects of surgery on ovarian tumors during pregnancy, particularly on the fetus, remain unknown, including those associated with anesthesia, surgical infections, and pneumoperitoneum. Opinions thus far on the safety of laparoscopic intervention during pregnancy for ovarian tumor have varied [3].

Some recent retrospective studies compared pregnancy outcomes after open and laparoscopic surgeries and reported no significant differences in neonatal outcome [4–9]. The SAGES guideline also states that laparoscopic surgeries are feasible at any time during pregnancy [8].

Therefore, in our department, laparoscopic surgery is performed under pneumoperitoneum even in pregnant cases if diagnosed as benign [7]. However, the uterine manipulator cannot be used to elevate the uterus during pregnancy. In addition, the enlarged pregnant uterus interferes with surgery for a tumor in the Cul-de-sac. In this case, the ovary lesion should be elevated with minimum stimulation to the pregnant uterus.

Murakami et al. reported using a metreurynter to elevate the ovary in a gasless surgery [10]. In our department, all laparoscopic surgeries are performed under pneumoperitoneum, both in pregnant and non-pregnant cases [6]. In pregnant cases, however, the enlarged uterus makes it challenging even to elevate the ovary tumor sunk in the Cul-de-sac. Lifting the ovaries with forceps while compressing the uterus can damage the pregnant uterus and cause heavy bleeding.

This report describes our method using a metreurynter in benign ovarian surgery during pregnancy. To the best of our knowledge, there have been no published reports of a large number of ovarian elevations using a metreurynter during pregnancy.

## Methods

We have performed laparoscopic surgery for 44 ovarian tumors during pregnancy to date. Among them, 11 cases involved a tumor found sunk in the Cul-de-sac and received ovarian cyst elevation using a metreurynter. The remaining 33 cases had tumors located in front of or over the uterus and had not received an ovarian cyst elevation. For the 11 patients who received an ovarian cyst elevation with a metreurynter, we evaluated the age, week of pregnancy, operative time, blood loss, tumor size, pathology, postoperative hospital stay, and success/failure of the elevation. Pregnancy outcomes and fetal status at delivery, including the week of gestation at delivery, delivery style, infant birth weight, Apgar score and obstetric complications were also evaluated. This study was approved by the Institutional Review Board of Kindai University Faculty of Medicine (No.RR01–29). All research was performed in accordance with Ethical Guidelines for Medical and Health Research Involving Human Subjects.

### Surgical technique

When an ovarian tumor is diagnosed by ultrasonography during pregnancy, magnetic resonance imaging (MRI) is performed for the differentiation of diagnosing benign and malignant lesions. This is the standard procedure in our hospital because MRI is considered to be more useful than ultrasonography in differentiation [11]. In our department, MRI is performed later than the 12th week of gestation. The optimal timing for laparoscopic surgery is between the 12th and 16th weeks of gestation when the placental and organ development stages have ended but the uterus has not grown large enough to impede the operative visual field [12].

**Fig. 1:**
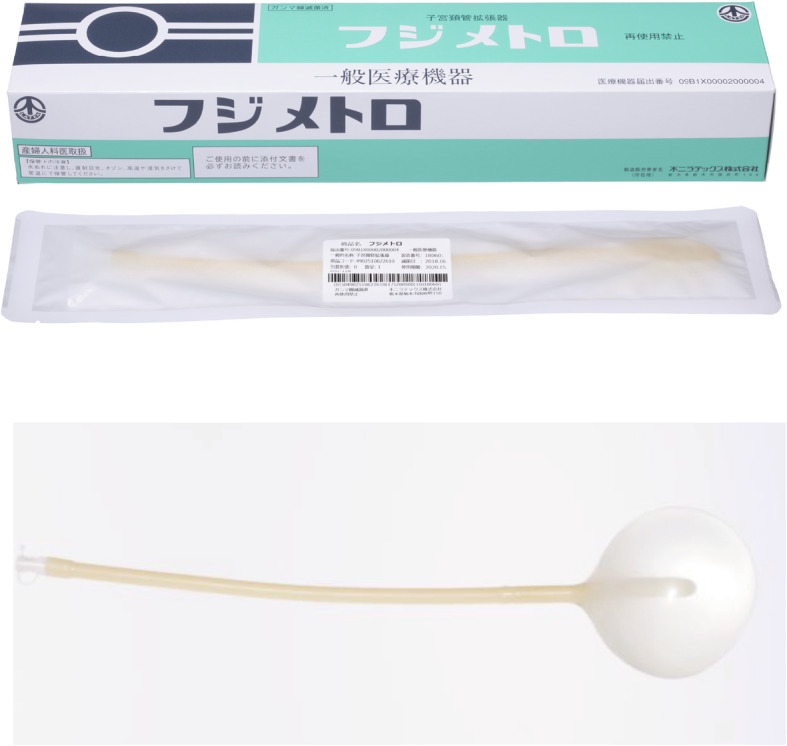
Metreurynter (Fujimetro®) used in our department. This figure was taken from the website of Fuji Latex Co

**Fig. 2:**
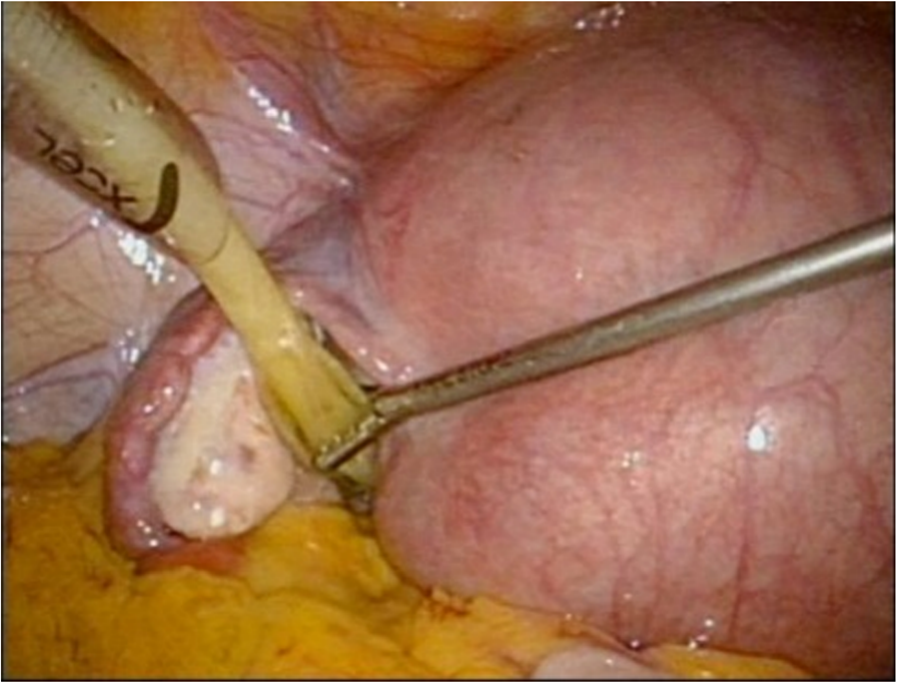
Ovarian cyst elevation using a metreurynter. A metreurynter is inserted via the 12-mm trocar

**Fig. 3:**
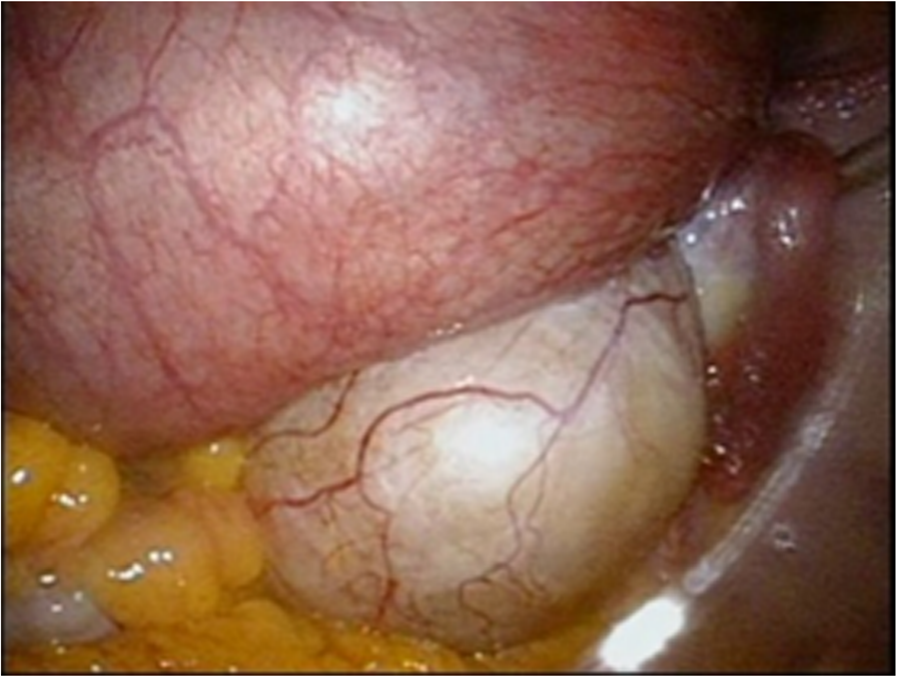
Saline (300 mL) injected in to the metreurynter in the Cul-de-sac

In our department, laparoscopy is performed by first attaining pneumoperitoneum through the umbilicus using a closed method and then inserting three 5-mm trocars, one in the median of the lower abdomen and one each to its right and left, and one 12-mm trocar through the umbilicus [7]. During pregnancy, all trocars are inserted more cranially than usual considering the enlarged uterus [13, 14]. At the start of the surgery, the patient is positioned head down at an angle of approximately 15°. A metreurynter (Fujimetro®, Fuji Latex Co., Tokyo, Fig. 1) is placed in the Cul-de-sac via the12-mm trocar through the left side of the uterus. An important tip at this point is to insert the metreurynter from around the lateral side of the tumor in the Cul-de-sac, targeting the margin below the tumor. The drawback of this method is the short 30-cm length of the metreurynter. This situation can be avoided by connecting an extension intravenous infusion line to the metreurynter to extend the length. After inserting the metreurynter is inflated with 300–500 mL of saline to elevate the tumor to the abdominal cavity (Figs. 2 and 3). At this point, transvaginal sonography is useful to ensure that the metreurynter is inserted correctly under the tumor. Once the ovaries are elevated, the deflated balloon is removed, and the metreurynter is then collected from the abdominal cavity. An ovarian tumorectomy is performed by making an incision halfway around the tumor along the equatorial line starting from the opposite side of the ovarian hilum, and the ovarian tumor alone is carefully removed to prevent cyst rupture [7]. The tumor is then transferred outside the body after its contents are aspirated inside an Endo Catch™ pouch (Medtronic Japan, Tokyo) inserted through the 12-mm trocar [7]. The patient is permitted to walk and eat on the day after surgery and is typically discharged 3–4 days after surgery. In our hospital, if the patient has no subjective symptom, tocolysis is not used and postoperative follow-up is managed in the same way as routine laparoscopic surgery.

## Results

Ovarian tumor elevation using a metreurynter was successful in nine cases, while it was unsuccessful in two cases in which we lifted the ovaries using forceps while compressing the uterus. Table 1 shows the surgical results. The success rate was 81.8% (9/11). The two unsuccessful cases had longer operative times. However, these cases included no bleeding or intraoperative complications.

**Table 1:** Surgical results of cases using a metreurynter

Case	Age range (years)	Body mass index	Week of pregnancy (week)	Operative time (min)	Blood loss (ml)	Tumor size (cm)	pathology	Postoperative hospital stay (day)	result
1	24–37	25.4	14	95	Small amount	5	Mature cystic teratoma	9	Success
2		18.4	16	105	Small amount	4	Mature cystic teratoma	7	Failure
3		19.3	15	105	37	6	Mature cystic teratoma	5	Success
4		17.0	16	72	Small amount	5	Mature cystic teratoma	4	Success
5		19.1	16	41	Small amount	7	Mature cystic teratoma	3	Success
6		20.7	16	110	50	9	Mature cystic teratoma	4	Failure
7		20.4	13	93	Small amount	10	Para ovarian cyst	4	Success
8		18.5	15	108	Small amount	7	Mature cystic teratoma	4	Success
9		23.0	15	107	Small amount	7	Mature cystic teratoma	4	Success
10		20.4	15	96	Small amount	6	Mature cystic teratoma	5	Success
11		24.8	13	100	Small amount	5	Mature cystic teratoma	5	Success

Table 2 shows the pregnancy prognoses. The pregnancy prognoses were favorable for both successful and unsuccessful cases. Case 8 and 9 involved a cesarean section for obstetric reasons, but the birth prognosis was favorable in all cases.

**Table 2:** Pregnancy prognosis of cases using a metreurynter

Case	Week of gestation at delivery (week)	Delivery style	Infant weight at delivery (g)	Apgar score (1 min/5 min)	Obstetric complication
1	38	Natural delivery	3090	8/9	
2	40	Natural delivery	2682	7/9	
3	40	Natural delivery	3484	8/9	
4	39	Natural delivery	2306	8/9	
5	39	Natural delivery	3210	8/9	
6	41	Natural delivery	3492	9/10	
7	41	Natural delivery	3838	9/10	
8	40	C-section	3195	9/9	cephalopelvic disproportion
9	40	C-section	3123	8/8	Fetal dysfunction
10	38	Natural delivery	3095	9/9	
11	During pregnancy				

## Discussion

The metreurynter is an obstetric device that is often used in Japan during delivery. Murakami et al. reported the usefulness of the instrument for ovarian cyst elevation in sling surgeries [10]. There have been several reports confirming the efficacy of a sling procedure to avoid the effects of general anesthesia and pneumoperitoneum [15]. However, other investigators have reported the need to convert to open surgery because of the limited field of view allowed by sling procedures [16]. Our institution uses pneumoperitoneum in all laparoscopic surgeries, and several recent studies reported no negative influence of pneumoperitoneum on neonatal outcomes [4–9]. In laparoscopic surgery for non-pregnant patients, the uterine manipulator can be used to elevate the lesion in the Cul-de-sac together with the uterus, and the method presented in this report may be useful particularly during pregnancy, when the uterine manipulator cannot be used. Other methods of elevating the ovary during pregnancy include the use of forceps to create space and of fingers to push up the Cul-de-sac from beneath. There are also reports on the use of a rectal probe (Rectal Sonde™ [Hakko Medical, Tokyo]) or SAND balloon catheter™ (Hakko Medical, Tokyo) to elevate the ovary [17]. However, SAND balloon catheter™ can only be used after the ovaries have been raised initially. In addition, the use of forceps accompanies the risk of hemorrhage, and the use of fingers and rectum probes may create considerable uterine pressure on the patient. In contrast, our method of using the metreurynter creates minimal pressure on the pregnant uterus. The metreurynter was originally designed to be sufficiently strong to be inserted into the pregnant uterus; hence, it had to be sufficiently strong. We experienced no metreurynter deflation in any of the cases evaluated in the present study.

The success rate of this technique for ovarian cyst elevation was 81.8% in this study. Transvaginal ultrasound was not combined in the introduction stage of this technique. In the two unsuccessful cases (Cases 2 and 6), the metreurynter inflated without confirming the position by transvaginal ultrasound and did not enter the Cul-de-sac despite repeated trials to elevate the ovary. Furthermore, the failure was not due to adhesion or other causes since these two cases had no prior surgical history.

All recent cases after Case 7 involved the use of transvaginal ultrasound to ensure that the metreurynter was swollen in the Cul-de-sac. No failure has occurred since the use of transvaginal ultrasound, thus indicating the importance and usefulness of this technique which ensures that the tip of the metreurynter is well placed in the Cul-de-sac.

Cases 1 and 2 were previous cases wherein tocolysis agents were administered postoperatively. Recent reports state, however, that it is not necessary to administer tocolysis agents after surgery [8, 18]. We allow patients to be discharged as in non-pregnancy cases.

## Conclusions

The metreurynter is an inexpensive and practical obstetric device, and its optimal use allows the performance of a procedure with minimal burden to the pregnant uterus. The procedure presented herein is likely to be a useful method for removal of a tumor found sunk in the Cul-de-sac. We recommend the use of this method to enable effective laparoscopic cystectomy of ovarian tumors during pregnancy.

## Data Availability

The datasets used and/or analysed during the current study available from the corresponding author on reasonable request.

## Abbreviation

MRI: Magnetic resonance imaging
